# Comparison of a directional cement delivery device versus conventional device in unilateral percutaneous kyphoplasty for the therapy of osteoporotic thoracolumbar fracture in the elderly

**DOI:** 10.1186/s13018-023-03506-0

**Published:** 2023-01-11

**Authors:** Xiaoji Zhou, Yongtao Liu, Xiaojian Cao, Genyang Jin, Hong Li

**Affiliations:** 1 Department of Orthopedics, The 904Th Hospital of PLA, Wuxi Clinical College of Anhui Medical University, 101 Xing Yuan Rd, Wuxi, 214044 China; 2 Department of Orthopedics, The Affiliated Hospital of Xuzhou Medical University, Xuzhou, Jiangsu Province China; 3 Department of Orthopedics, The First Affiliated Hospital of Nanjing Medical University, Nanjing, Jiangsu Province China

**Keywords:** Osteoporosis, Percutaneous kyphoplasty, Vertebral compression fracture, Unilateral puncture

## Abstract

**Background:**

Percutaneous kyphoplasty (PKP) has been demonstrated to be effective in the treatment of osteoporotic vertebral compression fractures (OVCF). However, bilateral puncture techniques take more time to accept more X-ray radiation; some spinal surgeons apply unilateral puncture PKP, but the cement cannot be symmetrically distributed in the vertebral body, so we apply a directional bone cement delivery device that undergoes PKP through the unilateral pedicle puncture. This research aims to compare the clinical and radiological results of PKP via unilateral pedicle approach using a traditional bone cement delivery device and a directional bone cement delivery device and determine the value of a directional delivery device for the therapy of thoracolumbar compression fracture in the elderly.

**Methods:**

We undertook a retrospective analysis of patients with single-level OVCF treated with unilateral pedicle puncture PKP from Jan 2018 to Jan 2020. Operation time, radiation exposure, bone cement injection volume, and the incidence of bone cement leakage were recorded for presentation, and the cement leakage and bone cement distribution were measured by X-ray and computed tomography scan. The patients were followed up postoperatively and were assessed mainly with regard to clinical and radiological outcomes.

**Results:**

There was no significant difference in the operation time, radiation exposure time, and incidence of bone cement leakage between the two groups. A significant difference was observed in the volume of bone cement injection between the two groups. All patients in both groups had significantly less pain after the procedures, compared with their preoperative period pain. There were no significant differences in Visual Analogue Scale, the relative height of the vertebral body, Cobb angle, and Quality of Life Questionnaire of the European Foundation for Osteoporosis between the two groups at 1 week after PKP, significant difference was observed only 12 months after operation.

**Conclusion:**

Application of directional bone cement delivery device is safe and feasible, compared with the application of traditional bone cement delivery device, without prolonging the operative time, radiation exposure time, and the incidence of bone cement leakage. It has the advantages of good short- and medium-term effect, excellent bone cement distribution, and low incidence of kyphosis recurrence.

## Background

Osteoporotic vertebral compression fractures (OVCF) are the most common type of osteoporotic fracture (brittle fracture), which are frequent in females older than 65 years. Nowadays, percutaneous kyphoplasty (PKP) is a widely used vertebral augmentation procedure to treat painful vertebral compression fractures and strengthen the stability of spine [1]. The conventional PKP requires a bilateral pedicle approach to create a symmetrical distribution of bone cement. However, bilateral pedicle puncture almost doubles the operation time and radiation exposure compared with a unilateral approach. Reducing operative time and radiation exposure of the doctor-patient is a valid objective. However, there is controversy about the efficacy of a unilateral pedicle approach. Some studies have reported similar short-term and long-term efficacy to bilateral procedures [2–5]. Conversely, another study suggested that unilateral PKP may lead to asymmetric distribution of bone cement and collapse of the contralateral side of the vertebral body under axial compression stress [6].

## Materials and methods

The study protocol was approved by the Institutional Review Board and the Ethics Committee of the 904 hospital of Chinese People Liberation Army.

### Patients

We performed a retrospective analysis of 221 patients with thoracic and lumbar compression fracture treated with unilateral pedicle puncture PKP.

Inclusion criteria include: ➀ aged more than 65 years; ➁ Bone attenuation on bone densitometry (*T* <  − 2. 5); ➂ severe back pain related to a single-level OVCF refractory to analgesic medication; ④ the affected vertebral body showed a hypointense signal on T1-weighted MR images and hyperintense signal on T2-weighted MR images. ⑤ Collapse 15% or more of the vertebral height.

Exclusion criteria included: ① secondary osteoporosis (corticosteroids, endocrine disorders and inflammatory process); ② abnormal coagulation mechanism; ③systemic or spine infection; ④ spinal metastatic cancer; ⑤ fractures involving all three columns of the vertebral body. ⑥ general poor physical health. Patients were divided into two groups according to bone cement delivery device: novel group (107 cases) using a directional bone cement delivery device; conventional group (114 cases) using a traditional bone cement delivery device.

### Surgical instruments

**Fig. 1:**
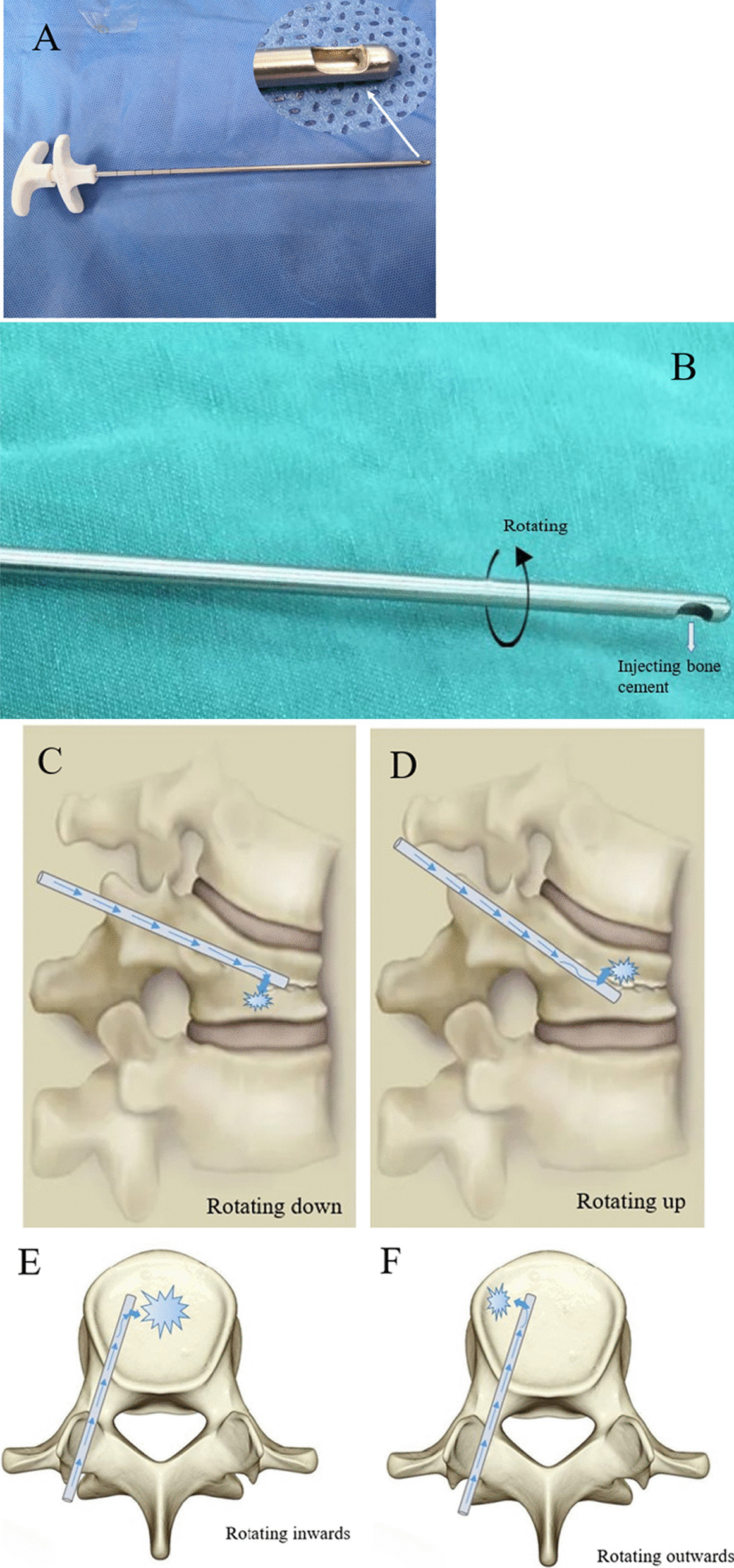
Schematic diagram of directional bone cement delivery device used in unilateral PKP. **A**, **B** The schematic diagram of directional bone cement delivery device and bone cement injected through the side opening. Cement filling direction can be controlled by rotating the device. The different rotating angles and injecting position of bone cement, for instance down (**C**), up (**D**), inward (**E**) and outward (**F**)

As shown in Fig. 1, the directional bone cement delivery device was designed on the basis of the traditional delivery device, which was 3.4 mm in diameter and 190 mm in length. The cannula was modified by sealing the front opening and creating a lateral opening in the distal end.

### Procedures

**Fig. 2:**
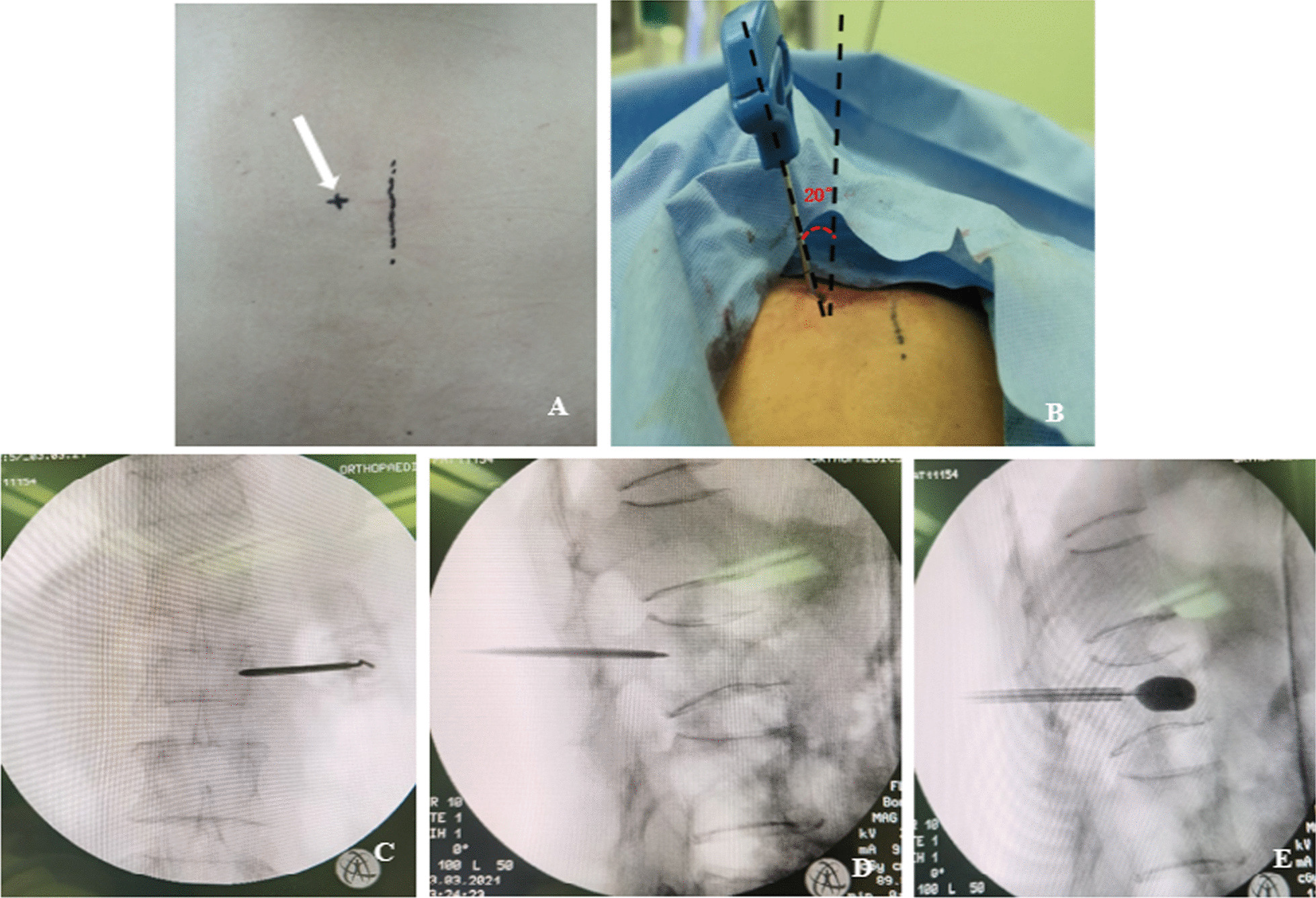
**A** Unilateral PKP skin incision design (white arrow represents the puncture point). **B** Puncture Angle range is 10°-15° (conventional group is 15°–20°). **C**, **D**, **E**, Puncture to establish a working channel, balloon expansion

All the unilateral PKP procedures were performed in the operating room under local anesthesia, and patients were placed prone, supported by two transverse bolsters under the thorax and pelvis. During the procedure, a unilateral transpedicular approach was adopted with the application of local anesthesia. The c-arm X-ray device was adjusted so that there was no bilateral shadow on the fractured vertebral body, and the shapes of the pedicles were symmetrical with the same distance to spinous process. The entry point in the vertebra was identified by fluoroscopy at the junction of the lateral edge of the pedicles and vertebral plate (2-o’clock position on the right side or at the 10-o’clock position on the left side). The extraversion angle was in the range from 10° to 15° (the conventional group was 15° to 20°) (Fig. 2). The trocar penetrated cortical bone at the lateral edge margin of the vertebral arch and was advanced medially and inferiorly. Lateral X-ray was used to confirm that the needle tip reached the posterior wall of the vertebral body. The needle was exchanged for a working cannula through which a drill trocar was advanced creating a channel for the balloon. Then, the drill trocar was removed and the inflatable balloon tamp was advanced into the anterior one-third of the affected vertebral body under fluoroscopy. The balloon was inflated under fluoroscopy no more than 200 psi. Polymethylmethacrylate (Tianjin Synthetic Material Industrial Research Institute Co., Ltd, Tianjin, China) was prepared with barium sulfate at room temperature (20℃) for about 5 min. And it was then injected manually into the cavity in the fractured vertebral body using a directional bone cement delivery device (conventional group using a traditional bone cement delivery device). The direction of bone cement injection can be adjusted by rotating the directional bone cement delivery device under fluoroscopy. All patients were advised to avoid extreme physical strain for 2 months.

### Outcome measures

**Fig. 3:**
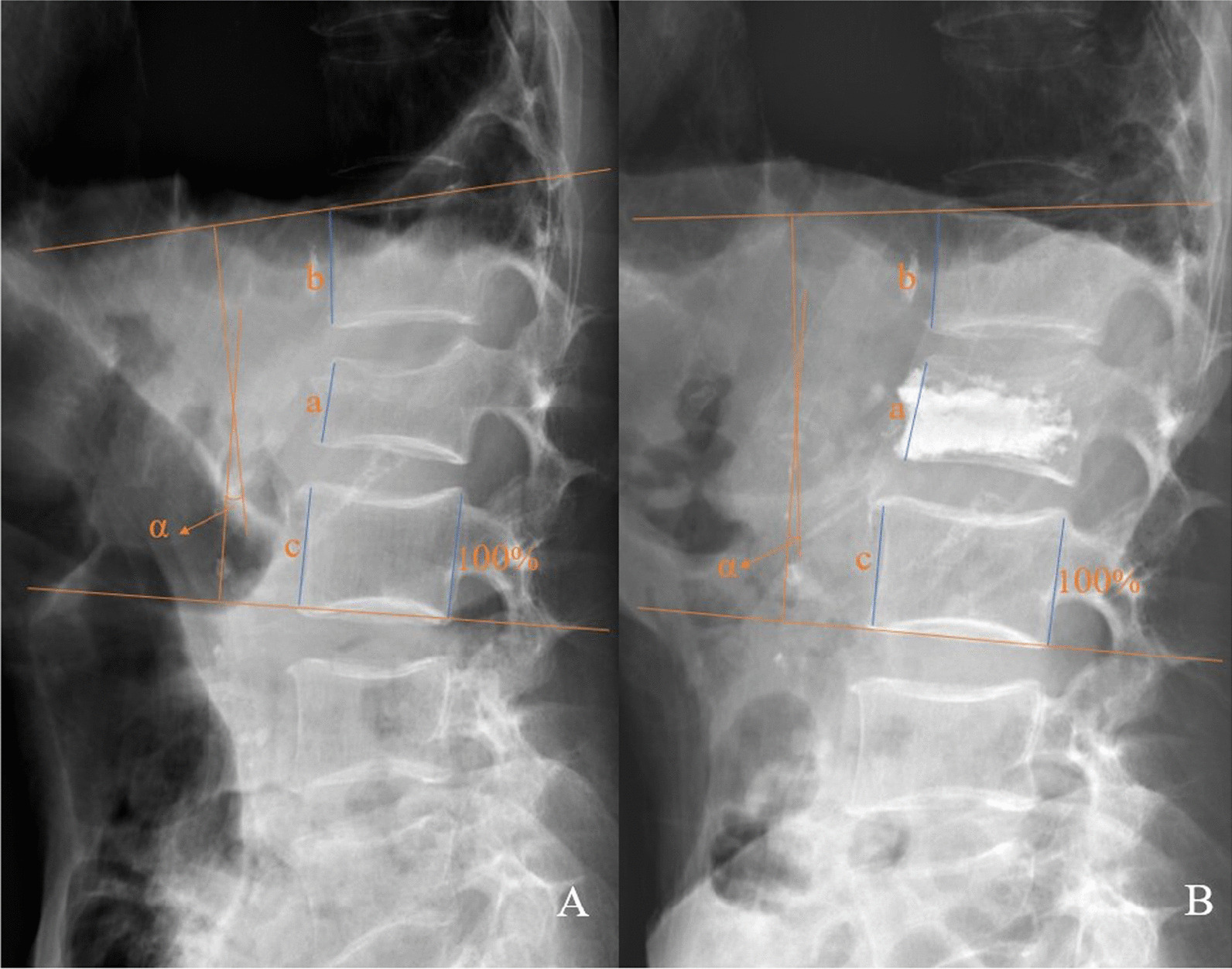
Relative anterior height (RAH) of the fractured vertebra and the kyphotic angle measurement method. Measurement of anterior height and kyphotic angle before (**A**) and after (**B**) operation. The posterior height (PH) of caudal healthy vertebra, which was adjacent to OVCF, was measured and transferred as 100% on the radiograph. TRA = *a*/[(*b* + *c*)/2]. The kyphotic angle (α) was measured as the angle between the superior endplate at one level above the injured vertebrae and inferior endplate at one level below the injured vertebrae

The operation time, radiation exposure time and the volume of bone cement injection were recorded for each patient in two groups. Clinical and radiographic assessments were evaluated before surgery, 1 week after surgery and 12 months after surgery. Radiographs and computed tomography (CT) scans were performed to assess the cement leakage and distributions of bone cement in the vertebral body. Anteroposterior and lateral standing radiographs were performed to measure the vertebral height and kyphotic angle of the vertebral body of all patients in three periods (preoperatively, 1 week after surgery, and 12 months after surgery). In the X-ray radiographs, the posterior height (PH) of the caudal healthy vertebra, which was adjacent to OVCF, was measured and transferred as 100% on the radiograph; then, using this scale, the anterior height (AH) index of the fractured vertebra and adjacent healthy vertebra were measured on the same radiograph (Fig. 3). And the relative anterior height (RAH) of the fractured vertebra was calculated according to the equation:

$${\text{RAH}} = {\text{fractured}}\;{\text{vertebral}}\;{\text{AH}}/\left[ {\left( {{\text{cranial}}\;{\text{healthy}}\;{\text{vertebra}}\;{\text{AH}} + {\text{caudal}}\;{\text{healthy}}\;{\text{vertebra}}\;{\text{AH}}} \right)/2} \right] { \times }100\%$$

The kyphotic angle (Cobb angle) was measured as the angle between the superior endplate at one level above the injured vertebra and inferior endplate at one level below the injured vertebra. Other possible local complications and adverse events were recorded (Fig. 3).

The pain was evaluated using a Visual Analogue Scale (VAS) from 0 (no pain at the base) to 10 (maximal imaginable pain at the summit). Quality of Life Questionnaire of the European Foundation for Osteoporosis (QUALEFFO) was investigated in all patients, which comprise a 41-item questionnaire organized into five domains (Pain, Physical Function, Social Function, General Health Perception, and Mental Function). Each domain’s score and QUALEFFO total scores were recorded on a 100-point scale, lower scores corresponding to better health-related quality of life.

**Fig. 4:**
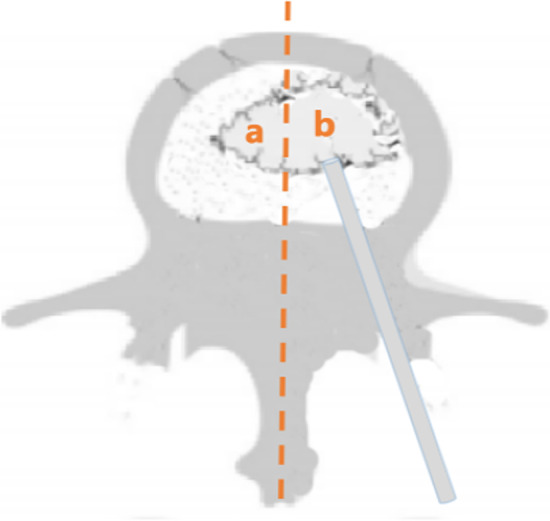
Measurement of bone cement distribution. Orange dotted line represents the midline of the vertebral body. Orange a represents the distribution of the bone cement on the non-puncture side. Orange b represents the distribution of the bone cement on the puncture side. *a*/(*a* + *b*) ≥ 10% is defined as excellent distribution; *a*/(*a* + *b*) < 10% is defined as poor distribution

According to the computed tomography scans of the injured vertebra, the bone cement distribution was analyzed using Image Pro-Plus 6.0 software (Media Cybernetics). Poor distribution: the area of bone cement exceeding the midline of the injured vertebra was ≤ 10% of the total area of bone cement, or the bone cement did not diffuse through the midline of the injured vertebra. Excellent distribution: the area of bone cement cross-filling the midline of the injured vertebra was > 10% of the total area of bone cement (Fig. 4).

### Statistical analysis

All statistical data were analyzed using SPSS software, version 26 (SPSS Inc, Chicago, IL). The baseline continuous variables were presented as mean ± standard deviation and compared by using independent two-sample t tests. Paired t tests were used to compare the preoperative and postoperative assessments in each group. The categorical variables were presented as number and percentage values and compared by using the χ2 and Fisher exact tests. A correlation analysis was applied for association with functional results compared with RAH and kyphotic angle. *P* < 0.05 was considered statistically significant.

## Results

**Fig. 5:**
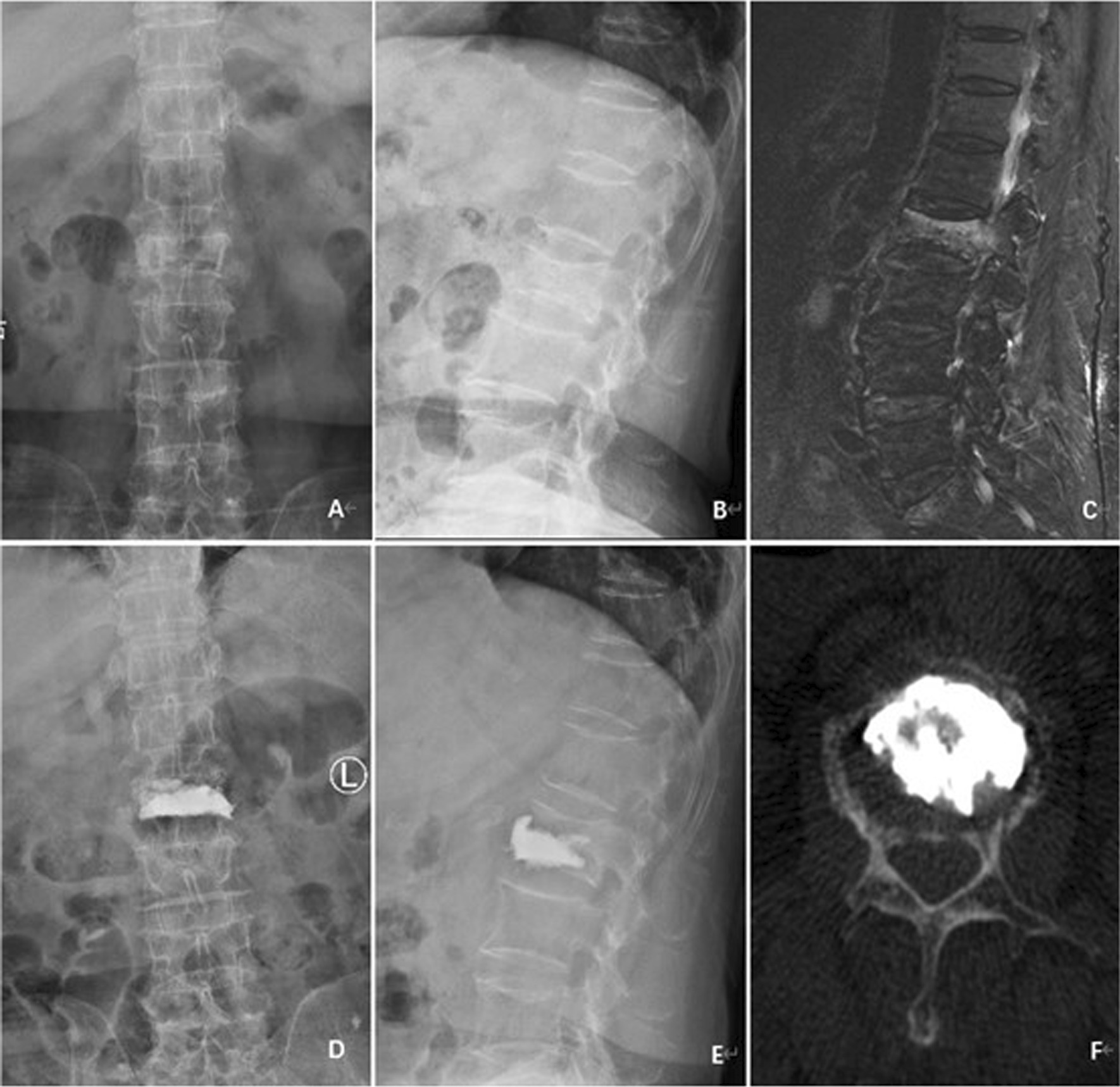
A 72-year-old female with single-level lumbar OVCF treated by unilateral PKP with directional bone cement delivery device. **A**, **B** Preoperative anteroposterior and lateral radiographs showed an L2 fracture. C, Sagittal T2W1 image showed the high signal within the vertebral body. **D**, **E** Postoperative anteroposterior and lateral radiographs showed an excellent bone cement distribution. **F** Postoperative CT scans showed that bone cement was mainly distributed symmetrically in the vertebral body

All surgical maneuvers were accurately performed under C-arm guidance in all cases, and no intraoperative deaths were reported in this study. The mean duration of follow-up was 16.2 months (range from 12 to 19 months). In terms of demographic data of patients, no significant difference was found between the two groups (Table 1). Typical cases are shown in Fig. 5.

**Table 1:** Characteristics of the study population

Characteristic	Novel Group	Conventional Group	Statistics(t/χ^2^)	*P*
Patients, no	107	114		
Age, mean (yr)	72.1 ± 4.1	71.4 ± 3.5	1.368	0.173
Females, no. (%)	75 (70.1)	79 (69.3)	0.017	0.898
Males, no. (%)	32 (29.9)	35 (30.7)		
BMD T score	− 3.0 ± 0.8	− 3.1 ± 0.6	1.055	0.292
Back pain persistent (days)	7 ± 3.1	6.6 ± 2.5	1.059	0.291
Injury site (cases)				
*T*_9_, *T*_10_, *T*_11_	30	32	0	1
*T*_12_, *L*_1_	48	51		
*L*_2_, *L*_3_, *L*_4_, *L*_5_	29	31		

### Intraoperative measurement

The mean radiation exposure time was not significantly different between the two groups, although a less time in conventional group (12.5 ± 3.1) S compared with the novel group (13.1 ± 2.8) S (Table 2). The operation time was (38.1 ± 4.1) min and (37.2 ± 3.5) min in the novel group and conventional group, respectively. The mean volume of the injected bone cement in the conventional group and novel group was (3.6 ± 0.3) ml and (4.9 ± 0.2) ml, respectively (*P* < 0.05).

**Table 2:** Comparison of operation time, radiation exposure time, volume of cement, and bone cement leakage between two groups

	Novel group	Conventional group	Statistics (t/χ^2^)	*P*
Operation time (min)	38.1 ± 4.1	37.2 ± 3.5	1.759	0.080
Radiation exposure time (S)	13.1 ± 2.8	12.5 ± 3.1	1.507	0.133
Volume of cement (ml)	4.9 ± 0.2	3.6 ± 0.3	37.651	0.000
Bone cement leakage, no. (%)	9 (8.4)	11 (9.6)	0.103	0.749

### Clinical results

**Fig. 6:**
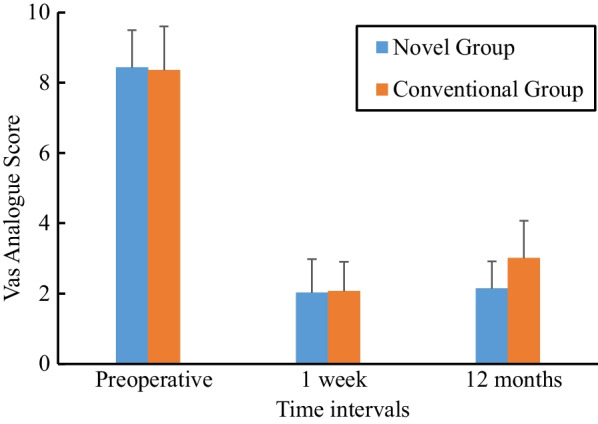
Preoperative and postoperative mean VAS scores for the two groups

The graph of the VAS score is shown in Fig. 6. All patients in both groups had significantly less pain after surgery, compared with their preoperative period. For the novel group, the mean VAS score decreased from 8.44 ± 1.06 before surgery to 2.02 ± 0.96 at 1 week postoperatively (*t* = 51.776, *P* = 0.000) and to 2.15 ± 0.76 at 12 months postoperatively (*t* = 55.058, *P* = 0.000). For the conventional group, the mean VAS score decreased from 8.36 ± 1.24 before surgery to 2.07 ± 0.83 at 1 week postoperatively (*t* = 55.165, *P* = 0.000) and to 3.01 ± 1.06 at 12 months postoperatively (*t* = 45.525, *P* = 0.000). No statistically significant differences were observed when VAS scores were compared between the two groups in the preoperative period and 1 week after surgery. However, statistically significant differences were found at 12 months after surgery (*t* = 6.892, *P* = 0.000).

**Fig. 7:**
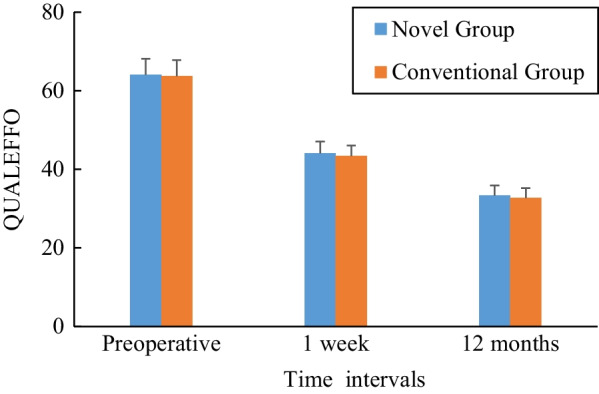
Preoperative and postoperative mean QUALEFFO scores for the two groups

According to QUALEFFO, five dimensionality concepts of pain, physical function, social function, general health perception, and mental function were measured, and the graph is shown in Fig. 7. All scores were significantly reduced in both groups after surgery; in the novel group, the mean score decreased from 64.07 ± 4.09 before surgery to 44.13 ± 2.92 at 1 week postoperatively (*t* = 39.175, *P* = 0.000) and to 33.35 ± 2.53 at 12 months postoperatively (*t* = 68.251, *P* = 0.000), and for the conventional group, the mean score decreased from 63.78 ± 4.00 before surgery to 43.43 ± 2.61 at 1 week postoperatively (*t* = 47.071, *P* = 0.000) and to 32.72 ± 2.48 at 12 months postoperatively (*t* = 73.855, *P* = 0.000). There were no statistically significant differences between the groups in terms of QUALEFFO at each assessment (*P* > 0.05).

### Radiological results

Preoperative and postoperative radiographical assessments of the two groups were measured and presented in Table 3. The RAH increased from (51.05 ± 6.08) % preoperatively to (54.5 ± 5.88) % at 1 week after operation and was (52.96 ± 5.02) % at 12 months after surgery in the novel group. Compared with the novel group, RAH increased from (49.61 ± 5.25) % preoperatively to (52.9 ± 6.21) % at 1-week post-operation and was (46.16 ± 4.55) % at 12 months postoperatively. There was no significant difference in the RAH index either preoperatively or at 1 week postoperatively in both groups. In addition, there were statistically significant differences between two groups at the 12-month follow-up (*P* < 0.05).

**Table 3:** Preoperative and postoperative radiographical assessment of the two groups

	Novel Group	Conventional Group	Statistics (t/χ^*2*^)	*P*
*RAH (%)*				
Preoperative	51.05 ± 6.08	49.61 ± 5.25	1.888	0.060
Postoperative 1 week	54.5 ± 5.88	52.9 ± 6.21	1.964	0.051
Postoperative 12 months	52.96 ± 5.02	46.16 ± 4.55	10.562	0.000
*Cobb (°)*				
Preoperative	20.25 ± 3.87	19.96 ± 3.05	0.621	0.536
Postoperative 1 week	16.08 ± 3.06	15.91 ± 3.69	0.372	0.711
Postoperative 12 months	17.24 ± 3.03	24.96 ± 4.01	16.068	0.000
*Bone cement distribution, no. (%)*				
Excellent distribution	87 (81.31)	74 (64.91)	7.502	0.006
Poor distribution	20 (18.69)	40 (35.09)		

**Fig. 8:**
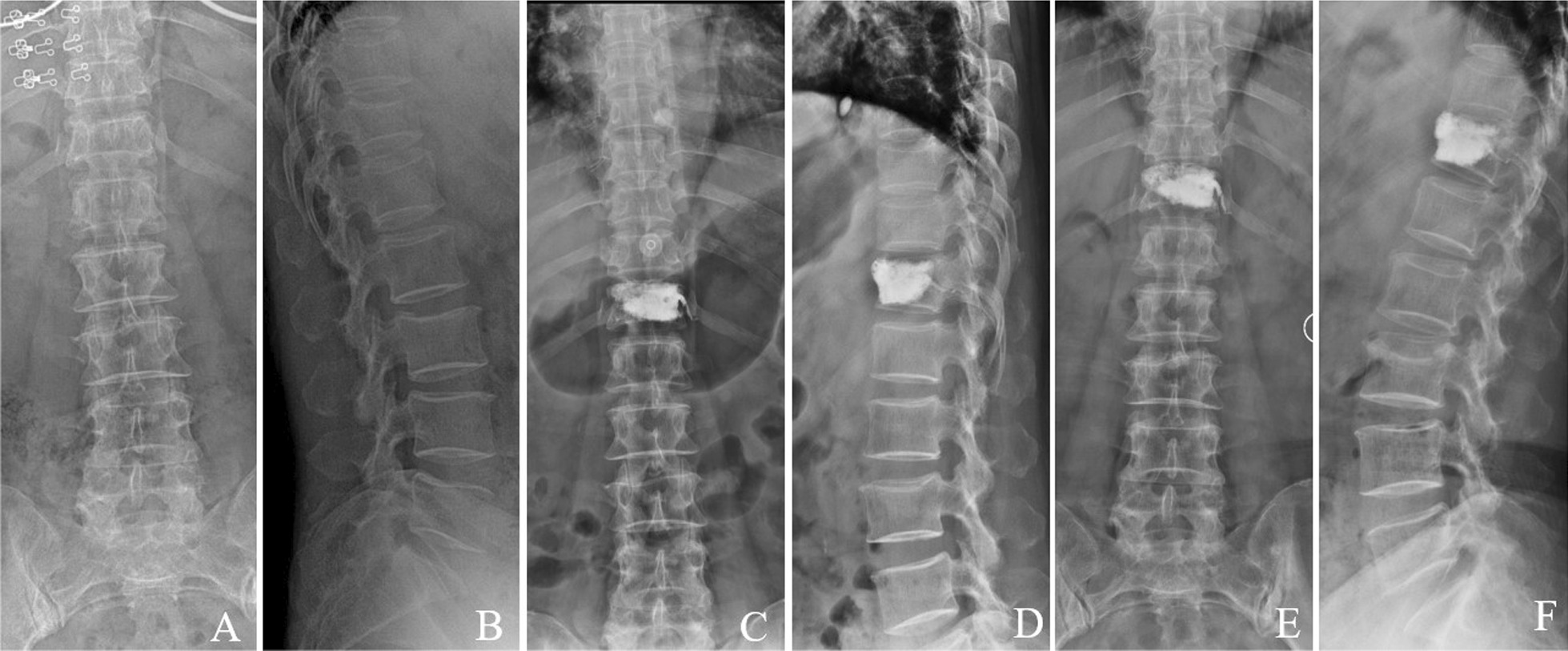
A 77-year-old female with single-level T_12_ OVCF treated by unilateral PKP with directional bone cement delivery device. **A**, **B** Preoperative anteroposterior and lateral radiographs showed a T_12_ fracture. **C**, **D**, one week after operation, the symmetrical distribution of bone cement. **E**, **F**, 12 months after operation: the bone cement remains symmetrically distributed. The anterior height of the injured vertebra and the kyphotic angle has not been changed

**Fig. 9:**
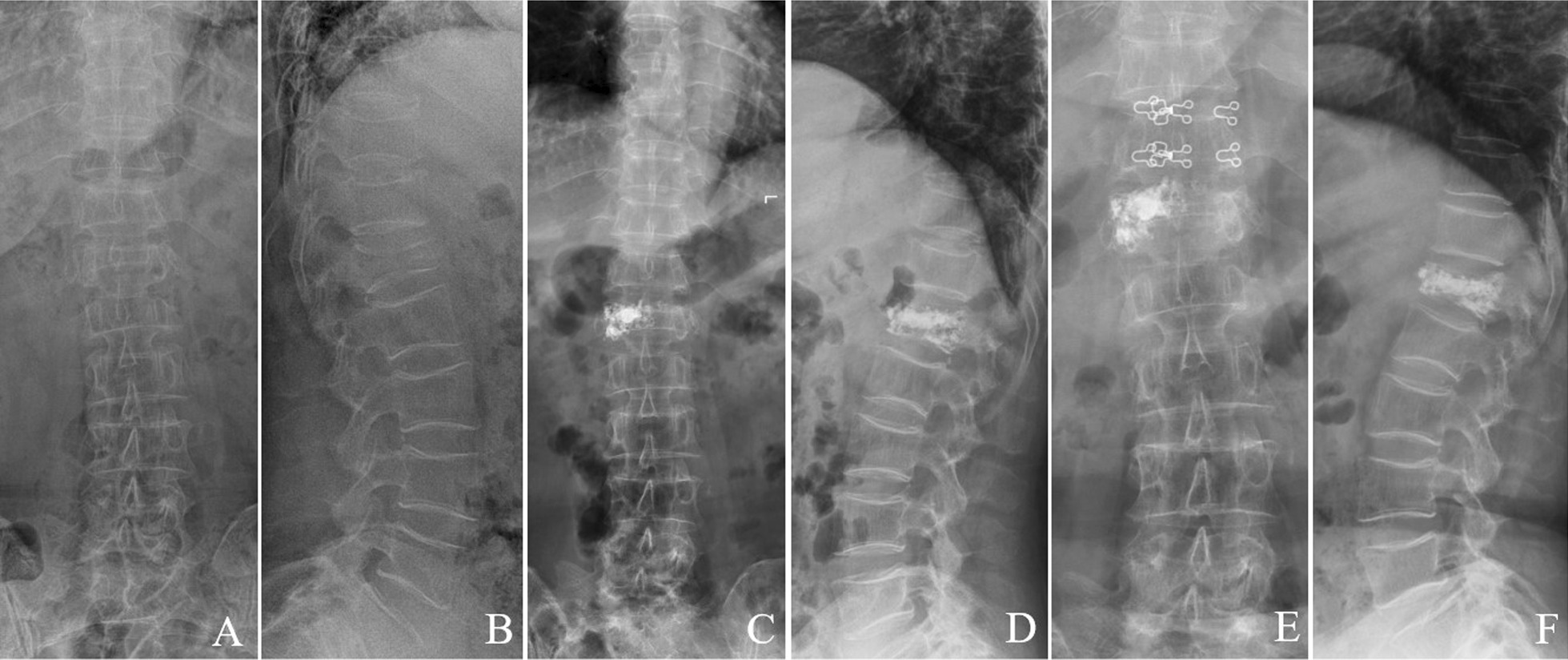
A 70-year-old female with single-level L_1_ OVCF treated by unilateral PKP with traditional bone cement delivery device. **A**, **B**, Preoperative anteroposterior and lateral radiographs showed a L_1_ fracture. **C**, **D**, one week after operation, the asymmetrical distribution of bone cement. **E**, **F**, 12 months after operation: the bone cement remains asymmetrically distributed. The anterior height of the injured vertebra has been diminished, and the kyphotic angle aggravated

The Cobb angle in the novel group ranged from (20.25 ± 3.87) ° before surgery to (16.08 ± 3.06) ° at 1 week after surgery and to (17.24 ± 3.03) ° at 12-month follow-up. In the conventional group, it changed from (19.96 ± 3.05) ° preoperatively to (15.91 ± 3.69) ° at 1 week after operation and to (24.96 ± 4.01) ° at 12 months after surgery. There was no significant difference between the two groups in Cobb angle immediately after surgery and at 1 week after surgery (*P* > 0.05). However, a statistically significant difference was observed between two groups at the 12-month follow-up, with the Cobb angle of the novel group being smaller than that of the conventional group (Table 3). Typical cases are shown in Fig. 8,9.

The improvement of QUALEFFO does not associate with improvement of RAH and kyphotic angle in both groups (*P* > 0.05). The correlation analysis of improvement of back pain VAS compared with radiological improvements (RAH and kyphotic angle) showed that both are associated with residual back pain. The kyphotic angle had a stronger correlation than the relative anterior height of the fractured vertebra. Pearson analysis of the clinical indexes and imaging parameters is summarized in Table 4.

**Table 4:** Pearson analysis of the clinical indexes and imaging parameters in both groups

	Novel Group				Conventional Group			
	△VAS		△QUALEFFO		△VAS		△QUALEFFO	
	*R*	*P*	*R*	*P*	*R*	*P*	*R*	*P*
△RAH	− 0.25	0.009	− 0.14	0.15	0.24	0.01	0.16	0.089
△Cobb	0.31	0.001	0.18	0.064	0.27	0.004	− 0.14	0.137

△ = last follow-up measurements-preoperative measurements

All postoperative computed tomographic scans were evaluated by an experienced radiologist who independently assessed bone cement distribution using the same evaluation standard. In the novel group, 87 of the 107 cases (81.31%) exhibited excellent cross-filling of bone cement, 20 cases (18.69%) exhibited poor cross-filling. In the conventional group, excellent cross-filling was 64.91%, and poor cross-filling was 35.09% (Table 3). Therefore, the bone cement distribution in the novel group was significantly better than that in the conventional group (*P* = 0.006).

### Complications

No procedure-related adverse events were observed in this study. Evaluation of intraoperative and postoperative radiographs revealed extra vertebral cement leakages in nine of 107 patients treated with directional bone cement delivery device (8.4%) and in 11 of 114 patients treated using traditional bone cement delivery device (9.6%) (*P* > 0.01). The site of cement leakage was the adjacent intervertebral disk through a cortical defect in eight cases (five cases in the novel group and three in the conventional group) and the paravertebral soft tissue via the segmental vein in 12 cases (five cases in the novel group and seven in the conventional group).

## Discussion

### Feasibility, safety, and clinical efficacy of unilateral PKP with directional bone cement delivery device

With the aging of the society, OVCF is the major health problem of older people, which dramatically increase morbidity and mortality [7]. Bilateral percutaneous kyphoplasty (PKP) is a traditional therapy for OVCF which bringing about a quick and lasting relief of pain as well as a durable correction of the spinal deformity. Recently, unilateral PKP is attracting increasing attention because of its advantages, including a shorter operative time, less trauma, and less radiation exposure to surgeons and patients [2, 8–10]. However, the distribution and amount of cement in the vertebral body by using unipedicular approach remain controversial. Some studies showed that poor efficacy of surgery could be caused by asymmetrical bone cement distribution and/or insufficient cement volume using unipedicular technique [11]. The relief of pain and the improvements of disability/QoL are largely associated with distribution of bone cement and the volume of cement in the vertebral body [6, 12, 13]. However, increasing the volume of bone cement injection may increase the incidence of cement leakage [14, 15]. Clinical studies [16, 17] suggested that 70% of symptomatic complications of vertebral cement augmentation were associated with the cement leakage. To overcome the shortcomings of unilateral PKP technology in the area of sufficient and symmetrical cement distribution, the directional bone cement delivery device was used to deliver bone cement multidirectional in the vertebral body. Due to the multi-directional injection, the injected cement is in the state of low-pressure diffusion, and subsequently, bone cement leakage caused by high pressure has been avoided. In this study, the cement distribution in the novel group was significantly better, and the volume of cement infusion was more than that in the conventional group even punctured through single side of the vertebral pedicle. Meanwhile, the incidence of cement leakage was not statistically increased in the novel group.

The opening side is located at the front end of the traditional delivery device; therefore, bone cement injected is mostly limited to the anterior side of the vertebral body. In traditional delivery device, in order to distribute bone cement evenly on both sides of the vertebral body via the unilateral puncture, the angle of trocar approach should be as oblique as possible without damaging the medial cortex of the pedicle, which may risk neurological damage. These problems are overcome by using the directional bone cement delivery device. In our study, the puncture angle in novel group was smaller than that in conventional group, and consequently makes the surgery safer.

QUALEFFO, one of the most widely used questionnaires of outcome measurement in patients with osteoporosis, has been shown to be valid and responsive to changes in functional status, and higher scores in this assessing system reveal worse physical function [18, 19]. In this study, both of two procedures markedly improved the physical function of patients, in addition to the immediate pain alleviation, which was reflected in an immediate and considerable change in VAS and QUALEFFO scores after the operation. Meanwhile, a significant improvement in the anterior height of vertebral body could be shown in both two groups postoperatively. This was also reflected in a significant pre- to postoperative improvement in Cobb angle.

### Medium-term efficacy of the two different bone cement delivery devices

Some studies showed that asymmetric distribution of bone cement in the vertebral body may lead to further uneven pressure-loading on the injured vertebral body, and thus causing collapse of the contralateral side of the vertebral body under axial compression stress [11, 19, 20]. Kim [21] as well demonstrated that uneven distribution of bone cement in the fractured vertebra body could cause the incomplete integration of bone cement cancellous bone, thus affecting the clinical efficacy. When using the directional bone cement delivery device, the cement can be directed flowing toward any area of vertebral body by rotating the device, mostly the center of the vertebral body, which a traditional one cannot, thereby permitting a more uniform strengthening effect of the whole fractured vertebra.

In this study, back pain VAS was significantly worse in the conventional group than the novel group at 12 months postoperatively. In addition, in the novel group, RAH and Cobb angle were significantly better than that in the conventional group at 12 months postoperatively. Back pain is correlated with the RAH and Cobb angle in the correlation analysis and may be affected in both groups especially by the Cobb angle. It seems likely that improvement of posttraumatic kyphosis can ease the residual back pain after operation in the medium-term. Improvements in QUALEFFO scores were observed in both surgical treatment methods and did not show a substantial difference between the groups at all time points. Physical function improvement is not correlated with the RAH and Cobb angle in the correlation analysis. This suggests that both two bone cement delivery devices can effectively improve physical function in the short-term and medium-term. The changes of RAH and Cobb angle do not have a significant effect on the improvement of physical function.

We find some limitations in this retrospective study. The number of patients included in this study may be relatively small; in addition, the 1-year follow-up period of the two groups was relatively shorter. Further long-term follow-up studies with a larger patient population are needed to generalize our results.

## Conclusion

This study confirmed that both directional delivery device and traditional delivery device can effectively lessen short-term back pain in patients with OVCF in unilateral PKP. The directional bone cement delivery device demonstrated better short- and medium-term efficacy, compared with the traditional one. Furthermore, directional bone cement delivery device can more effectively improve the filling of bone cement in the vertebral body, restore the height of the vertebral body, and improve kyphosis without prolonging the surgical time, adding radiation exposure, increasing puncture angle, and increasing the incidence of bone cement leakage. In conclusion, directional bone cement delivery device is a good choice for treating painful OVCF in unilateral PKP.

## Data Availability

All data generated during this study is included in this published article.

## Abbreviations

PKP: Percutaneous kyphoplasty
OVCF: Osteoporotic vertebral compression fractures
CT: Computed tomography
PH: Posterior height
AH: Anterior height
RAH: Relative anterior height
VAS: Visual Analogue Scale
QUALEFFO: Quality of Life Questionnaire of the European Foundation for Osteoporosis
BMD: Bone Mineral Density

## References

[1] Klazen CA, Lohle PN, de Vries J (2010). Vertebroplasty versus conservative treatment in acute osteoporotic vertebral compression fractures (Vertos II): an open-label randomised trial. Lancet.

[2] Papadopoulos EC, Edobor-Osula F, Gardner MJ (2008). Unipedicular balloon kyphoplasty for the treatment of osteoporotic vertebral compression fractures: early results. J Spinal Disord Tech.

[3] Steinmann J, Tingey CT, Cruz G (2005). Biomechanical comparison of unipedicular versus bipedicular kyphoplasty. Spine (Phila Pa 1976).

[4] Yan L, Jiang R, He B (2014). A comparison between unilateral transverse process-pedicle and bilateral puncture techniques in percutaneous kyphoplasty. Spine (Phila Pa 1976).

[5] Rebolledo BJ, Gladnick BP, Unnanuntana A (2013). Comparison of unipedicular and bipedicular balloon kyphoplasty for the treatment of osteoporotic vertebral compression fractures: a prospective randomised study. Bone Joint J.

[6] Liebschner MA, Rosenberg WS, Keaveny TM (2001). Effects of bone cement volume and distribution on vertebral stiffness after vertebroplasty. Spine (Phila Pa 1976).

[7] Rousing R, Hansen KL, Andersen MO (2010). Twelve-months follow-up in forty-nine patients with acute/semiacute osteoporotic vertebral fractures treated conservatively or with percutaneous vertebroplasty: a clinical randomized study. Spine (Phila Pa 1976).

[8] Wang S, Wang Q, Kang J (2014). An imaging anatomical study on percutaneous kyphoplasty for lumbar via a unilateral transverse process-pedicle approach. Spine (Phila Pa 1976).

[9] Tohmeh AG, Mathis JM, Fenton DC (1999). Biomechanical efficacy of unipedicular versus bipedicular vertebroplasty for the management of osteoporotic compression fractures. Spine (Phila Pa 1976).

[10] Liu MX, Xia L, Zhong J (2020). Is it necessary to approach the compressed vertebra bilaterally during the process of PKP?. J Spinal Cord Med.

[11] Chen B, Li Y, Xie D (2011). Comparison of unipedicular and bipedicular kyphoplasty on the stiffness and biomechanical balance of compression fractured vertebrae. Eur Spine J.

[12] Sun H, Li C (2016). Comparison of unilateral and bilateral percutaneous vertebroplasty for osteoporotic vertebral compression fractures: a systematic review and meta-analysis. J Orthop Surg Res.

[13] Lin J, Qian L, Jiang C (2018). Bone cement distribution is a potential predictor to the reconstructive effects of unilateral percutaneous kyphoplasty in OVCFs: a retrospective study. J Orthop Surg Res.

[14] Wang C, Fan S, Liu J (2014). Basivertebral foramen could be connected with intravertebral cleft: a potential risk factor of cement leakage in percutaneous kyphoplasty. Spine J.

[15] Kinzl M, Benneker LM, Boger A (2012). The effect of standard and low-modulus cement augmentation on the stiffness, strength, and endplate pressure distribution in vertebroplasty. Eur Spine J.

[16] Hatzantonis C, Czyz M, Pyzik R (2017). Intracardiac bone cement embolism as a complication of vertebroplasty: management strategy. Eur Spine J.

[17] Hoppe S, Wangler S, Aghayev E (2016). Reduction of cement leakage by sequential PMMA application in a vertebroplasty model. Eur Spine J.

[18] Lips P, Cooper C, Agnusdei D (1999). Quality of life in patients with vertebral fractures: validation of the Quality of Life Questionnaire of the European Foundation for Osteoporosis (QUALEFFO). Working Party for Quality of Life of the European Foundation for Osteoporosis. Osteoporos Int.

[19] Zhou C, Li Q, Huang S (2016). Validation of the simplified Chinese version of the quality of life questionnaire of the European foundation for osteoporosis (QUALEFFO-31). Eur Spine J.

[20] Zhang LG, Gu X, Zhang HL (2015). Unilateral or bilateral percutaneous vertebroplasty for acute osteoporotic vertebral fracture: a prospective study. J Spinal Disord Tech.

[21] Kim DJ, Kim TW, Park KH (2010). The proper volume and distribution of cement augmentation on percutaneous vertebroplasty. J Korean Neurosurg Soc.

